# Combined Treatment of Xenon and Hypothermia in Newborn Rats - Additive or Synergistic Effect?

**DOI:** 10.1371/journal.pone.0109845

**Published:** 2014-10-06

**Authors:** Hemmen Sabir, Lars Walløe, John Dingley, Elisa Smit, Xun Liu, Marianne Thoresen

**Affiliations:** 1 Neonatal Neuroscience, School of Clinical Sciences, University of Bristol, Bristol, United Kingdom; 2 Department of Physiology, Institute of Basic Medical Sciences, University of Oslo, Oslo, Norway; 3 College of Medicine, Swansea University, Swansea, United Kingdom; 4 Department of General Pediatrics, Neonatology and Pediatric Cardiology, Heinrich-Heine-University Düsseldorf, Düsseldorf, Germany; University of Naples Federico II, Italy

## Abstract

**Background:**

Breathing the inert gas Xenon (Xe) enhances hypothermic (HT) neuroprotection after hypoxia-ischemia (HI) in small and large newborn animal models. The underlying mechanism of the enhancement is not yet fully understood, but the combined effect of Xe and HT could either be synergistic (larger than the two effects added) or simply additive. A previously published study, using unilateral carotid ligation followed by hypoxia in seven day old (P7) rats, showed that the combination of mild HT (35°C) and low Xe concentration (20%), both not being neuroprotective alone, had a synergistic effect and was neuroprotective when both were started with a 4 h delay after a moderate HI insult. To examine whether another laboratory could confirm this finding, we repeated key aspects of the study.

**Design/Methods:**

After the HI-insult 120 pups were exposed to different post-insult treatments: three temperatures (normothermia (NT) NT_37°C_, HT_35°C_, HT_32°C_) or Xe concentrations (0%, 20% or 50%) starting either immediately or with a 4 h delay. To assess the synergistic potency of Xe-HT, a second set (n = 101) of P7 pups were exposed to either HT_35°C_+Xe_0%_, NT+Xe_20%_ or a combination of HT_35°C_+Xe_20%_ starting with a 4 h delay after the insult. Brain damage was analyzed using relative hemispheric (ligated side/unligated side) brain tissue area loss after seven day survival.

**Results:**

Immediate HT_32**°**C_ (p = 0.042), but not HT_35**°**C_ significantly reduced brain injury compared to NT_37°C_. As previously shown, adding immediate Xe_50%_ to HT_32°C_ increased protection. Neither 4 h-delayed Xe_20%_, nor Xe_50%_ at 37**°**C significantly reduced brain injury (p>0.050). In addition, neither 4 h-delayed HT_35**°**C_ alone, nor HT_35°C_+Xe_20%_ reduced brain injury. We found no synergistic effect of the combined treatments in this experimental model.

**Conclusions:**

Combining two treatments that individually were ineffective (delayed HT_35°C_ and delayed Xe_20%_) did not exert neuroprotection when combined, and therefore did not show a synergistic treatment effect.

## Introduction

Therapeutic hypothermia (HT) has been shown to be safe and reduces brain injury after hypoxia ischemia (HI) in human newborns [1], [2], [3], [4], [5], [6] and animal models [7], [8]. Since 2010, HT has become standard clinical treatment after perinatal asphyxia [9]. Outcome data show that HT has the potential to improve long term outcome [10], [11], [12]. However, 50% of cooled newborns still suffer death or severe disability [12], [13], [14] and improved neuroprotective treatment is sought. In theory, such additional treatment might have either an additive or a synergistic effect with HT.

We and others have previously shown in different animal species that the noble gas xenon (Xe) alone is neuroprotective after hypoxia-ischemia [8], [15], [16], and that neuroprotection is increased when combined with HT [7], [8], [17], [18]. However, the exact mechanism by which Xe is neuroprotective is not yet fully understood. Xenon is an anesthetic and is often referred to as an “ideal anesthetic” as it has been shown to be safe, providing rapid onset and offset characteristics with no adverse hemodynamic or other effects [19]. Xenon is not only neuroprotective, but has also been shown to be cardio- and nephroprotective [20], [21], [22].

When combining two treatments, one needs to document the potency of the combination, as drugs administered during the neonatal period may have toxic effects on the newborn body and brain. Two treatments combining to give an effect greater than the sum of the two treatment effects alone are synergistic, where as an additive effect is the sum of two individual treatments. There are opposing views on whether combining Xe and HT demonstrates a synergistic or an additive effect. We have previously shown in neonatal rats and newborn pigs suffering hypoxia-ischemia, that both Xe and HT have individual neuroprotective abilities and that their combined neuroprotection is additive [7], [8]. In contrast, Ma and colleagues have found that the neuroprotective effects of Xe and HT are synergistic [17], [23]. However, they have used a different setup to ours to prove this synergistic effect. Therefore, this current study was carried out to examine the potential synergistic effect of Xe and HT in newborn rats using an experimental setup with their design, which has previously suggested a synergistic effect between Xe and HT [17].

## Materials and Methods

### Procedures

All procedures were carried out under Home Office license in accordance with UK regulations and approved by the University of Bristol’s animal ethical review panel.

### Animal Experiments

#### 1. Control Experiments of added neuroprotection by Xe-HT

The first set of experiments was set out to verify our previously published finding of additive neuroprotection between 50% Xe and HT at 32°C (HT_32°C_) in this neonatal animal model, [7] using hemispheric area loss after 1 week survival as the outcome measure [24].

**Figure 1:**
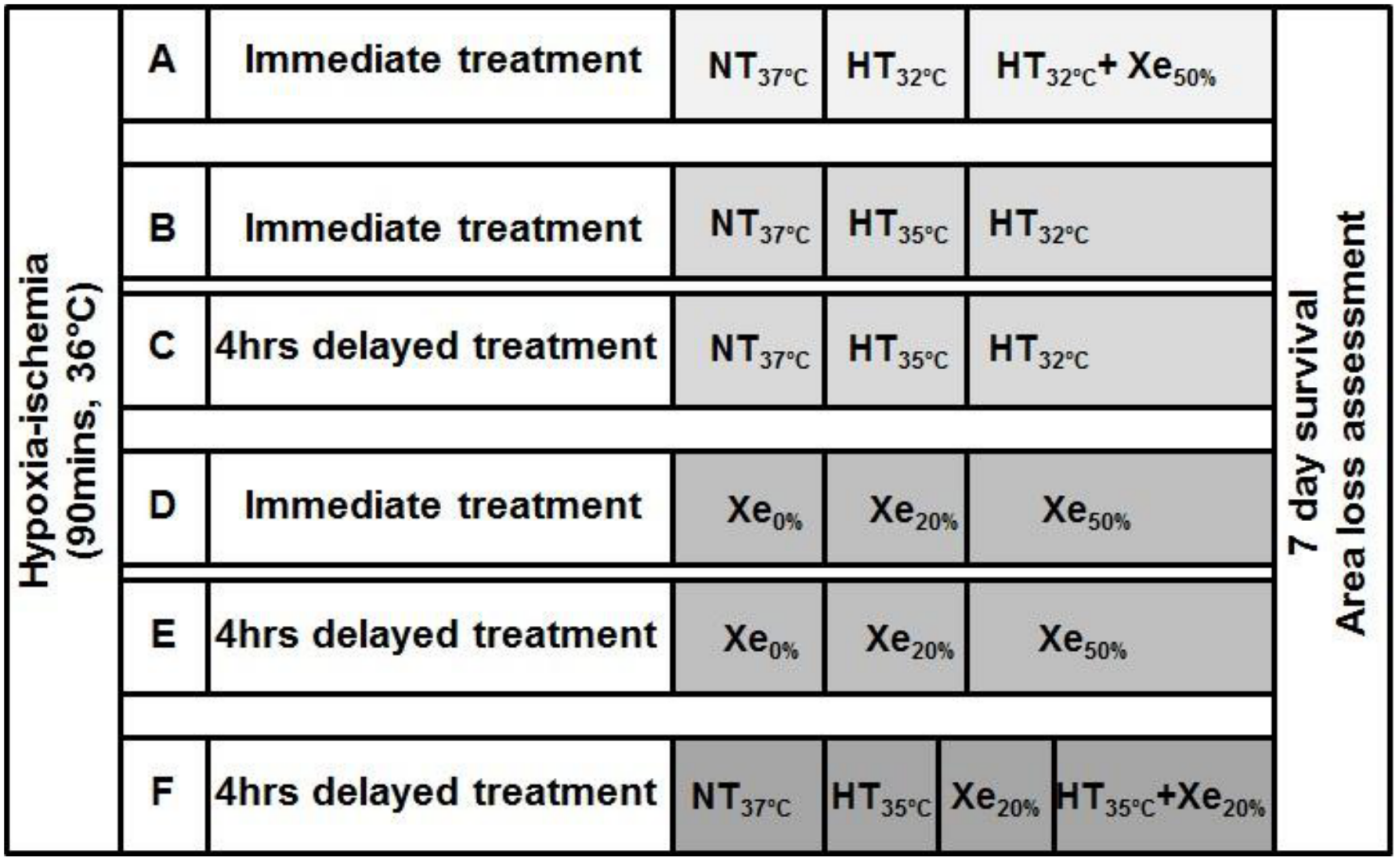
Experimental design for different treatment strategies.

Forty-five P7 Wistar rat pups of both sexes underwent a left common carotid ligation under general anesthesia as previously described [24]. After a maximum recovery of 180 mins, whilst pups were with their dams, pups were exposed to 8% oxygen for 90 min at a rectal temperature (T_rectal_) of 36.0°C in a temperature-controlled chamber, resulting in a moderate hypoxic-ischemic (HI) insult with ∼40% brain area loss. This HI insult has been shown to be of the same severity as that of the original paper by Hobbs *et al.* [7], [24]. The temperature of the animals was continuously recorded by animals carrying a rectal (IT-21, Physitemp Instruments, Clifton, New Jersey, USA) or skin (CritiCool, MTRE, Charter Kontron Ltd, Milton Keynes, UK) temperature probe. Animals carrying a temperature probe were excluded from further analysis, as they respond differently to hypoxia ischemia [25]. Subsequently after the HI surviving pups from different litters were randomized equally to each group, and matched for sex and weight (Figure 1A).

A total of 38 pups survived the insult (7 died). Six probe animals were excluded from further analysis and 32 pups were randomized to 3 treatments lasting 5 h starting immediately: normothermia at 37°C (NT_37°C_, n = 11), HT at 32°C (HT_32°C_, n = 10) or combined HT at 32°C with 50% Xe (HT_32°C_+Xe_50%_, n = 11). The temperature was continuously measured in the additional “probe animals” in each chamber with a rectal temperature probe and a skin probe on the abdomen. Both probes were calibrated to ±0.1°C over a range of 20.0 to 40.0°C against a certified mercury-in-glass thermometer (BS593, Zeal, London, UK). The temperatures were maintained with a servo-controlled mat (CritiCool, MTRE, Charter Kontron Ltd, Milton Keynes, UK). Rectal temperature correlates within 0.1°C with brain temperature in P7 rats [26]. For the HT groups, a T_rectal_ of 32.0°C±0.2°C was achieved within 15 mins. The stable Xe concentration was achieved and measured within the chamber using our previously described closed re-circulating system to conserve Xe [27]. The set Xe concentration (20% or 50%) was achieved within 15 mins and maintained, for the 5 h treatment period. After the treatment period, pups were immediately removed from the chamber and returned to their dams. No clinical seizures were observed. While with their dam, the temperature of the probe animals was intermittently measured. All animals were kept in a 12∶12 h dark/light cycle at 22°C environmental temperature with adequate food and water, and weights were checked daily until the end of the survival period at P14.

#### 2. Different Temperatures and Xenon Concentrations with and without delay

The second set of experiments was performed to assess the effect of different temperatures (Part 1) or different Xe concentrations (Part 2) on brain area loss at one week survival. During the different treatments the temperature was continuously measured in additional “probe animals” as described above.

Part1: 96 P7 pups of both sexes from 10 litters underwent a moderate HI insult as described above. If untreated, the NT group, this insult results in ∼40% relative hemispheric area loss after one week. [24], [26] Three pups died during the insult and 12 pups were excluded from further analysis as they carried a temperature probe. Pups were randomized to 6 groups and exposed either immediately for 5 h in 21% oxygen at HT_32°C_ (n = 14), HT_35°C_ (n = 13) or NT_37°C_ (n = 14) or after a 4 h delay: HT_32°C_ (n = 13), HT_35°C_ (n = 13) or NT_37°C_ (n = 14) (Figure 1B–C). During the 4 h delay pups were kept with their dams. For the HT groups, a T_rectal_ of 32.0°C±0.2°C or 35.0°C±0.2°C was achieved within 15 minutes. After the treatment period, pups were immediately removed from the chamber and returned to their dams until P14.

Part 2: 99 P7 pups of both sexes from 10 litters underwent the moderate HI insult as described above. Five pups died during the insult and 12 pups carrying a temperature probe were excluded. Pups were randomized to 6 groups to be exposed either immediately for 5 h in 21% oxygen at NT to different Xe concentrations: Xe_0%_ (n = 14), Xe_20%_ (n = 14) or Xe_50%_ (n = 14) or after a 4 h delay: Xe_0%_ (n = 13), Xe_20%_ (n = 14) or Xe_50%_ (n = 13) (Figure 1D–E). After the treatment period, pups were immediately removed from the chamber and returned to their dams until P14.

#### 3. Combined Experiments of Different Temperatures and Xenon Concentrations with and without delay

The third set of experiments was carried out to assess whether the combination of mild hypothermia (HT_35°C_) and a low Xe concentration (Xe_20%_) is neuroprotective when started with a 4 h delay.

One hundred and one P7 pups of both sexes from 20 litters underwent a left common carotid ligation under general anesthesia. All pups were exposed to 8% oxygen for 90 min at T_rectal_ of 36.0°C in a temperature-controlled chamber. Three animals died during the insult and 16 probe animals were excluded from further analysis, leaving 82 pups for randomization matched for litter, sex and weight (Figure 1F) to 4 groups. After a 4 h delay with the dam, pups underwent 5 h of NT_37°C_ (n = 21), HT_35°C_ (n = 20), NT_37°C_+Xe_20%_ (n = 20) or HT_35°C_+Xe_20%_ (n = 21). After the 5 h treatment period, pups were returned to their dam and survived until P14.

### Histopathology and Area Measurement

After seven days of survival, transcardiac perfusion with 10% neutral-buffered formalin was performed under deep isoflurane/N_2_O-anesthesia and brains kept in 10% neutral-buffered formalin until further processing. After cutting coronal 3 mm blocks through the brain, using a standard matrix for uniformity (ASI Instruments Inc., Warren, Michigan, USA), brains were embedded in paraffin. Blocks were cut for 5 µm sections and stained with hematoxylin and eosin (H&E). Two sections from each of two neighboring blocks representing cortex, hippocampus, basal ganglia and thalamus, were scanned (Epson, Perfection V30, Telford, UK) with 1200 dpi resolution. As previously described, [24] brain area tissue loss was measured by an individual blinded to the experimental treatment using ImageJ software (ImageJ, version 1.43, National Institutes of Health, USA). The midline of each brain section was identified on the image and the brain divided by its hemispheres (left vs right). ImageJ was used to measure the area of viable tissue in the left and right hemisphere. The ratio of the measured brain area was calculated for the two sections per brain and the average percentage of area loss was calculated (1–(Area Ratio (right vs left))×100). This method of area loss assessment has been shown to correlate well with our validated neuropathology score [24].

### Data Analysis

Statistical analyses were performed with SPSS version 18 (SPSS Inc., Chicago, IL). For two-group comparisons the t-test was used. One way ANOVA was used to compare the different treatment groups. To assess a possible effect of sex and weight of pups on brain area loss and to assess combined effects of the two treatments (HT and Xe), linear regression analysis was used. Two-sided testing with *p*<0.05 was considered statistically significant. Descriptive data are presented as mean ± standard deviation (±SD).

## Results

### 1. Control Experiments of added neuroprotection by Xe-HT

**Figure 2:**
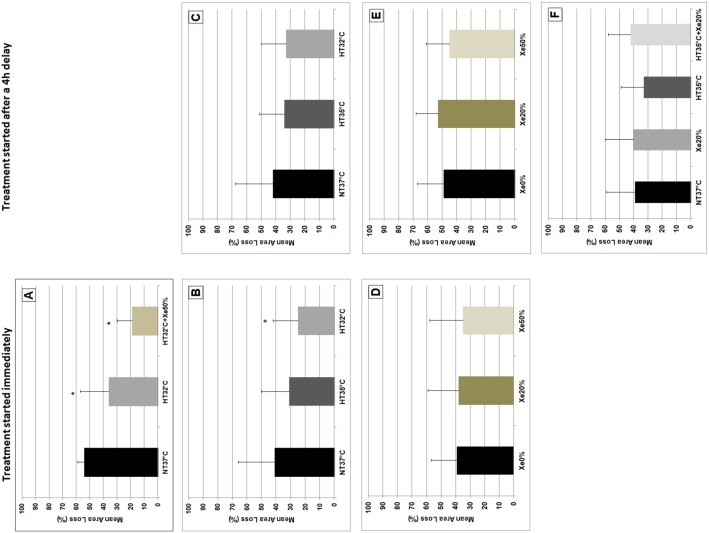
Shows the mean brain area loss (+SD) of each of the performed experiments, either being immediately after the hypoxic-ischemic insult or after a 4 h delay. (A) Showing significant neuroprotection of hypothermia (HT_32°C_) compared to normothermia (NT_37°C_) (p = 0.02) and additional neuroprotection in the combination of 50% xenon with hypothermia (HT_32°C_+Xe_50%_) (p<0.001). (B+C) Shows a significant reduction of mean brain area loss in animals being immediately cooled to 32°C after hypoxia-ischemia, compared to animals being normothermic (p = 0.04). Immediate hypothermia at 35°C (HT_35°C_) did not significantly reduce brain area loss (p = 0.26). Starting the treatment with a 4 h delay showed no significant neuroprotection of both hypothermia temperatures compared to the normothermia group (p>0.05). (D+E) Shows that immediately after hypoxia ischemia neither 20% (Xe_20%_) nor 50% xenon (Xe_50%_) significantly reduce brain area loss compared to the group treated in air (Xe_0%_) (p>0.05). Starting the treatment with a 4 h delay did not change the results and showed a similar pattern (p>0.05). (F) Shows the results of the combined treatment started with a 4 h delay after hypoxia-ischemia. None of the treatment groups significantly reduced brain area loss compared to the NT_37°C_ group. The combination of HT_35°C_ and Xe_20%_ started with a 4 h delay did not reduce brain area loss.

When repeating our previously published experiments and using brain area loss instead of a neuropathology score as an outcome parameter, we found that mean (±SD) area loss in the NT_37°C_ group was 54.2% (±5.56). There was a significant reduction in mean brain area loss in the immediately cooled animals (HT_32°C_, 36.0% (±21.83), p = 0.015) compared to the NT_37°C_ group. Also, as previously shown, adding Xe_50%_ to HT_32°C_ significantly reduced mean brain area loss (19.9% (±11.32) compared to NT_37°C_ (p<0.001) and HT_32°C_ alone (p = 0.045), ANOVA p<0.001)) (Figure 2A). This shows that we were able to confirm our findings of additional neuroprotection combining 50% Xe with HT at 32°C, using brain area loss at 1 week survival as the outcome.

### 2. Different Temperatures and Xenon Concentrations with and without delay

In this part of the study we found that immediate HT_32°C_ significantly reduced mean brain area loss when compared to NT_37°C_ (HT_32°C_ 25.8% (±17.37) vs NT_37°C_ 41.6% (±25.38), p = 0.042). Immediate HT_35°C_ did not significantly reduce brain area loss, when compared to NT_37°C_ (HT_35°C_ 31.5% (±19.00) vs NT_37°C_, p = 0.272, (Figure 2B). The ANOVA did not show any significant difference between the treatment groups (p = 0.163).

After a 4 h delay, neither HT_32°C_ nor HT_35°C_ significantly reduced brain area loss when compared to NT_37°C_ (HT_32°C_ 33.1% (±17.65) vs NT_37°C_ 42.2% (±26.85), p = 0.327 and HT_35°C_ 34.1% (±17.36) vs NT_37°C_ 42.2% (±26.85), p = 0.382) (Figure 2C). The ANOVA did not show any significant difference among the treatment groups (p = 0.502).

In the second part of the study we found that immediate Xe_20%_ did not significantly reduce mean brain area loss when compared to animals treated in air (Xe_20%_ 38.4% (±21.42) vs Xe_0%_ 39.7% (±18.43), p = 0.871). In addition, immediate Xe_50%_ did not significantly reduce mean brain area loss when compared to animals treated in air (Xe_50%_ 35.5% (±23.97) vs Xe_0%_ 39.7% (±18.43), p = 0.618) (Figure 2D). In addition the ANOVA did not show significant differences between the groups (p = 0.872).

After a 4 h delay neither Xe_20%_, nor Xe_50%_ significantly reduced mean brain area loss when compared to animals treated in air (Xe_20%_ 53.2% (±15.60) vs Xe_0%_ 49.7% (±18.78), p = 0.624 and Xe_50%_ 45.8% (±16.11) vs Xe_0%_ 49.7% (±18.78), p = 0.589) (Figure 2E). The ANOVA did not show any significant difference between the treatment groups (p = 0.560).

Table 1 shows that there was no significant difference between the different treatment groups regarding sex and weight at P7. In addition linear regression did not show a significant effect of sex and weight on brain area loss in any of the groups.

**Table 1:** Mean (± SD).

A	**Immediate Treatment**	**NT_37°C_**	**HT_32°C_**	**HT_32°C_+Xe_50%_**
	N (male)	11 (6)	10 (5)	11 (4)
	Weight at P7 [g]	15.46 (±1.50)	14.84 (±1.32)	15.61 (±1.12)
	Weight gain at P14 [g]	5.95 (±3.47)	7.84 (±3.37)	6.16 (±5.21)
B	**Immediate Treatment**	**NT_37°C_**	**HT_35°C_**	**HT_32°C_**
	N (male)	14 (6)	13 (5)	14 (5)
	Weight at P7 [g]	16.02 (±1.25)	15.93 (±1.99)	15.54 (±1.59)
	Weight gain at P14 [g]	14.82 (±2.91)	14.53 (±2.48)	12.81 (±3.45)
C	**4 h delayed Treatment**	**NT_37°C_**	**HT_35°C_**	**HT_32°C_**
	N (male)	14 (7)	13 (9)	13 (8)
	Weight at P7 [g]	15.78 (±1.41)	16.17 (±2.19)	16.37 (±1.76)
	Weight gain at P14 [g]	14.27 (±2.51)	14.70 (±2.14)	14.47 (±2.13)
D	**Immediate Treatment**	**Xe_0%_**	**Xe_20%_**	**Xe_50%_**
	N (male)	14 (7)	14 (6)	14 (7)
	Weight at P7 [g]	15.14 (±2.09)	14.48 (±1.59)	14.80 (±1.88)
	Weight gain at P14 [g]	14.20 (±2.08)	12.88 (±2.78)	14.51 (±2.27)
E	**4 h delayed Treatment**	**Xe_0%_**	**Xe_20%_**	**Xe_50%_**
	N (male)	13 (8)	14 (7)	13 (5)
	Weight at P7 [g]	17.34 (±1.32)	16.64 (±1.44)	16.32 (±1.40)
	Weight gain at P14 [g]	13.52 (±2.88)	13.89 (±2.03)	13.62 (±1.92)
F	**4 h delayed Treatment**	**NT_37°C_**	**NT_37°C_+Xe_20%_**	**HT_35°C_**
	N (male)	21 (10)	20 (9)	20 (10)
	Weight at P7 [g]	13.83 (±2.66)	14.24 (±1.24)	13.94 (±1.50)
	Weight gain at P14 [g]	8.52 (±2.67)	7.99 (±3.09)	8.27 (±2.21)

There was no significant difference between the treatment groups regarding sex, weight at 7-days of age (P7) or weight gain at 14-days of age (P14). Experimental setup (A–E) as described in Figures 1+2.

### 3. Combined Experiments of Different Temperatures and Xenon Concentrations with and without delay

In the combined experiments, we found that none of the different treatment groups had a significantly reduced mean brain area loss, compared to the NT_37°C_ group. Mean (±SD) area loss was 39.7% (±20.88) for the NT_37°C_ group, 40.1% (±20.91) for the NT_37°C_+Xe_20%_ group (p = 0.905) and 33.2% (±16.90) for the HT_35°C_ group (p = 0.310). When combining both treatments (HT_35°C_+Xe_20%_) mean (±SD) brain area loss was 42.4% (±16.62), which was not significantly different from the NT_37°C_ group (p = 0.599) (Figure 2F). The ANOVA did not show any significant difference between the treatment groups (p = 0.594). In the regression analysis, the coefficient of the interaction term between cooling and Xe was small and non-significant.

Combining the treatments, Xe_20%_ and NT_37°C_, that individually were not effective was not neuroprotective indicating that there was no synergistic effect between the two treatments.

There was no significant difference between the different treatment groups regarding sex and weight at P7 (Table 1). In addition linear regression did not show a significant effect of sex and weight on brain area loss in any of the groups.

### 4. Animal Model Variability

**Figure 3:**
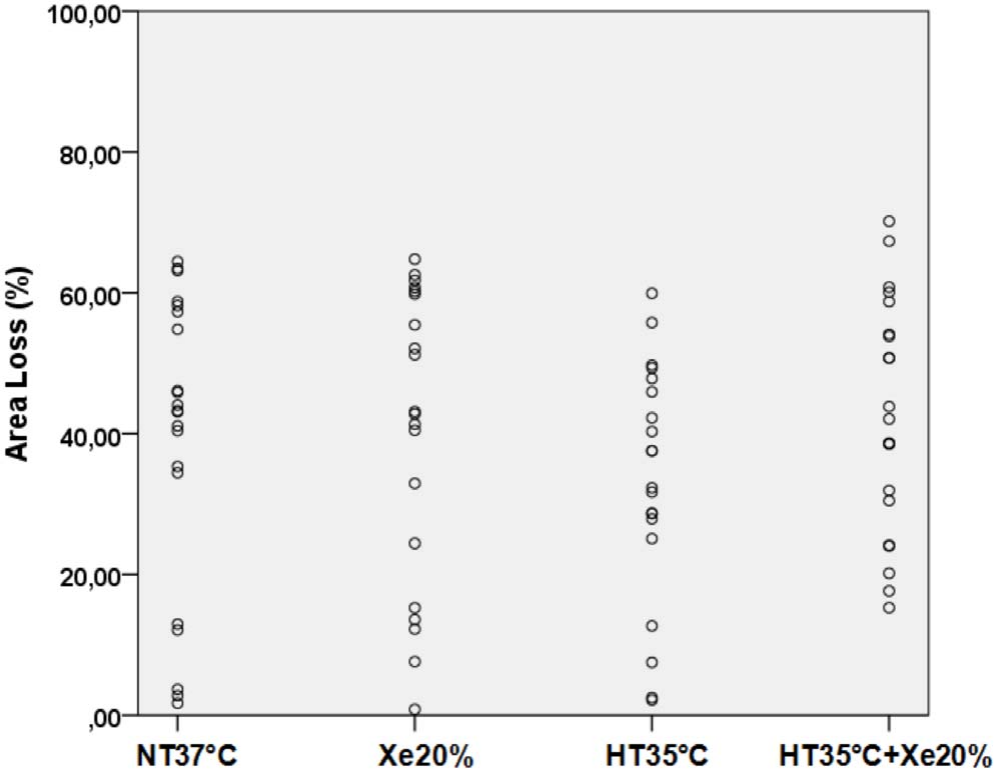
Shows the large variability in mean area loss within each group from the 4 h delayed combination experiments. Each circle (o) represents one of the animals from the individual groups used for analysis.

Figure 3 shows the scatter plots of percentage area loss for the four groups from the combined experiments of different temperatures and Xe concentrations. It shows the variability in injury pattern in this animal model within each treatment group, which is well known [7], [24], [28], [29], [30], [31]. This variability has been observed throughout all performed experiments and therefore explains the large standard deviations within each treatment group and necessitates the need for large numbers in each group.

## Discussion

The main finding of this study is that combining two treatments (HT_35°C_ and Xe_20%_), both not individually neuroprotective, did not result in neuroprotection. We were unable to replicate the results of a previously published study [17], despite using the same animal model, treatment temperature, Xe concentration and delay before start of treatment. We recognize that a significant weakness of the model is the variability of injury within treatment groups (with associated large standard deviations), therefore we used larger group sizes. However, we were unable to show a synergistic effect between the two treatments (HT_35°C_+Xe_20%_).

To verify our own previous findings [7] we first showed that the immediate combined treatment of HT_32°C_+Xe_50%_ significantly reduced relative brain area loss, compared to HT_32°C_ alone. We have previously shown that brain area loss correlates well with our previously used global pathology score [24]. It is a quick, reliable and reproducible way to assess brain injury in this animal model as long as the groups are large. Rice and Vannucci introduced the unilateral carotid ligation “rat hypoxia-ischemia-model” in 1981 [30]. In their original paper they used hemispheric brain weight ratio (ligated/non-ligated side) to assess brain injury. Many authors initially used this weight ratio in their studies [32], [33], [34]. As it is technically not very easy to cut an injured brain in the midline, it has been stated that brain weight assessment can be used for screening purposes only [35] and histopathology will remain the gold standard. We have validated our brain area loss from histological section versus pathology scoring [24].

As shown in Figure 3 and mentioned in many other papers [7], [24], [28], [29], [30], [31] the variability in degree of injury in this animal model is large. Standard deviation typically is ∼20%, which necessitates the need for large groups. Nevertheless this experimental fact strengthens the translational value of the model, as it reproduces variability observed in human neonatal hypoxia-ischemia [29].

Xenon has been shown to offer additional neuroprotection when combined with HT after hypoxia-ischemia in newborn rats and pigs [7], [8], [18]. However, whether this effect is additive or synergistic remains to be fully elucidated. Ma and colleagues have previously suggested that the effect of combining the two treatments is synergistic [17], [23]. In contrast, we have found no evidence of synergy, but only additive effects when the two treatments were combined both in rats and newborn pigs [7], [8]. In our previous papers we did not use the same temperatures and Xe concentrations as Ma *et al*., so we have replicated those conditions here. In addition to the *in* *vivo* work we have repeated in this paper, their conclusion about synergy is partly based on cell culture work [17]. Ma *et al.* present results obtained from *in* *vitro* cultures of neuronal and glial cells exposed to experimental ischemia in the form of oxygen-glucose deprivation for 75 minutes, followed by a 16-hour recovery period. During the oxygen-glucose deprivation and the recovery period, different cultures were exposed to five different temperatures from 37°C to 20°C, or five different concentrations of Xe in the atmosphere from 0% to 75%, alone and in combination. The amount of injury was quantified by the release of lactate dehydrogenase (LDH). LDH release decreased with decreasing temperature and with increasing concentrations of Xe, showing that both interventions protected the neuronal cells in this cell culture model. Using results obtained from using 12.5% Xe and 33°C as their example, the authors argue that the combination acts synergistically. This conclusion is based on an application of isobolographic analysis [36]. However, there are considerable problems both with both the application of isobolographic analysis to this kind of data, as well as the assumption that the method is appropriate in the first place. One general problem is that the degree of HT (relative to 37°C) is treated as if it were similar to the concentration of a drug. However, there is one important difference. The concentration of a drug can never have a value below zero, while the degree of hypothermia may well be negative (for temperatures below 37°C). In addition, when the dose-effect relationship of the individual agents (temperature and Xe) has a different maximum, as they have in the reported experimental results, the isobole of additivity is not a straight line, but a downwards curve [37]. Furthermore the 50% of maximum response points appear to not have been correctly plotted in their Figure 1G. The 12.5% Xe point should perhaps be moved to the right to about 17%, and the other point should perhaps be moved vertically from 4°C to about 6°C. These two corrections to their Figure 1G, a downward movement of the middle section of the curved isobole of additivity and a movement upwards and to the right of the experimental points, will likely bring the experimental points well within a 95% confidence band around the curved isobole of additivity. Our conclusion is therefore that no synergistic effect of temperature and Xe has been shown in these cell culture experiments.

However, in their paper, they also present results from *in* *vivo* experiments, which they claim give direct evidence of a synergistic effect between HT and Xe [17]. As our previous experiments have suggested an additive, rather than synergistic, effect, we have repeated the experimental conditions described by Ma *et al.* Like many other researchers in this field, they used the Rice and Vannucci rat model to produce unilateral brain injury. Brain weight ratio was used as their outcome parameter seven days after the insult.

Considering that a raw screening tool (brain weight) was used as an outcome parameter, it is interesting to note both the small number of rats in each treatment group (n = 6–8), and that statistically significant differences were obtained. This would suggest very small standard deviations within the data [17]. Most error bars in their figures are presented as standard error of the mean, being 7% or less. This would correspond to standard deviations less than 20%. These standard deviations are substantially smaller than what we, and many other researchers, report in the same experimental model [7], [24], [29], [30]. For this reason, and because we have not observed any indication of synergistic effects between HT and Xe in our own experiments, we wanted to repeat the experiments following their experimental protocol, but using a higher number of animals in each group. Larger group numbers were also required according to our own statistical power calculations.

There are some limitations to our study. First, Ma et *al.* have also shown improvement of functional outcome thirty days after HI [17]. Our animals only survived 1 week. We have however shown in a previous study, where animals were randomized to short (7d) or long (30d) survival, that neuropathology after 7d correlated strongly with both 30d pathology and functional outcome in this animal model [7]. It is very important to study the underlying mechanisms of the evolution of brain injury and neuroregeneration. This will require a different design with a sequence of survival times.

We did not find any evidence for a synergistic effect of HT and Xe, when using the experimental protocol of Ma et *al.* With a 4 h delay, none of the different individual treatments were effective, nor was the combined treatment of HT_35°C_+Xe_20%_. This suggests that these two conditions do not provide a synergistic effect when combined.
