# Correction: Combined Treatment of Xenon and Hypothermia in Newborn Rats - Additive or Synergistic Effect?

**DOI:** 10.1371/journal.pone.0115986

**Published:** 2014-12-15

**Authors:** The PLOS ONE Staff

There is an error in the sixth to last sentence of the fourth paragraph of the Discussion. The correct sentence should read: The concentration of a drug can never have a value below zero, while the degree of hypothermia may well be negative (for temperatures above 37°C).

There is an error in the third to last sentence of the “Combined Experiments of Different Temperatures and Xenon Concentrations with and without delay” section of the Results. The correct sentence should read: Combining the treatments, Xe_20%_ and NT_35°C_, that individually were not effective was not neuroprotective indicating that there was no synergistic effect between the two treatments.

There is an error in Figure 2. The authors have provided the corrected figure below.

**Figure 2:**
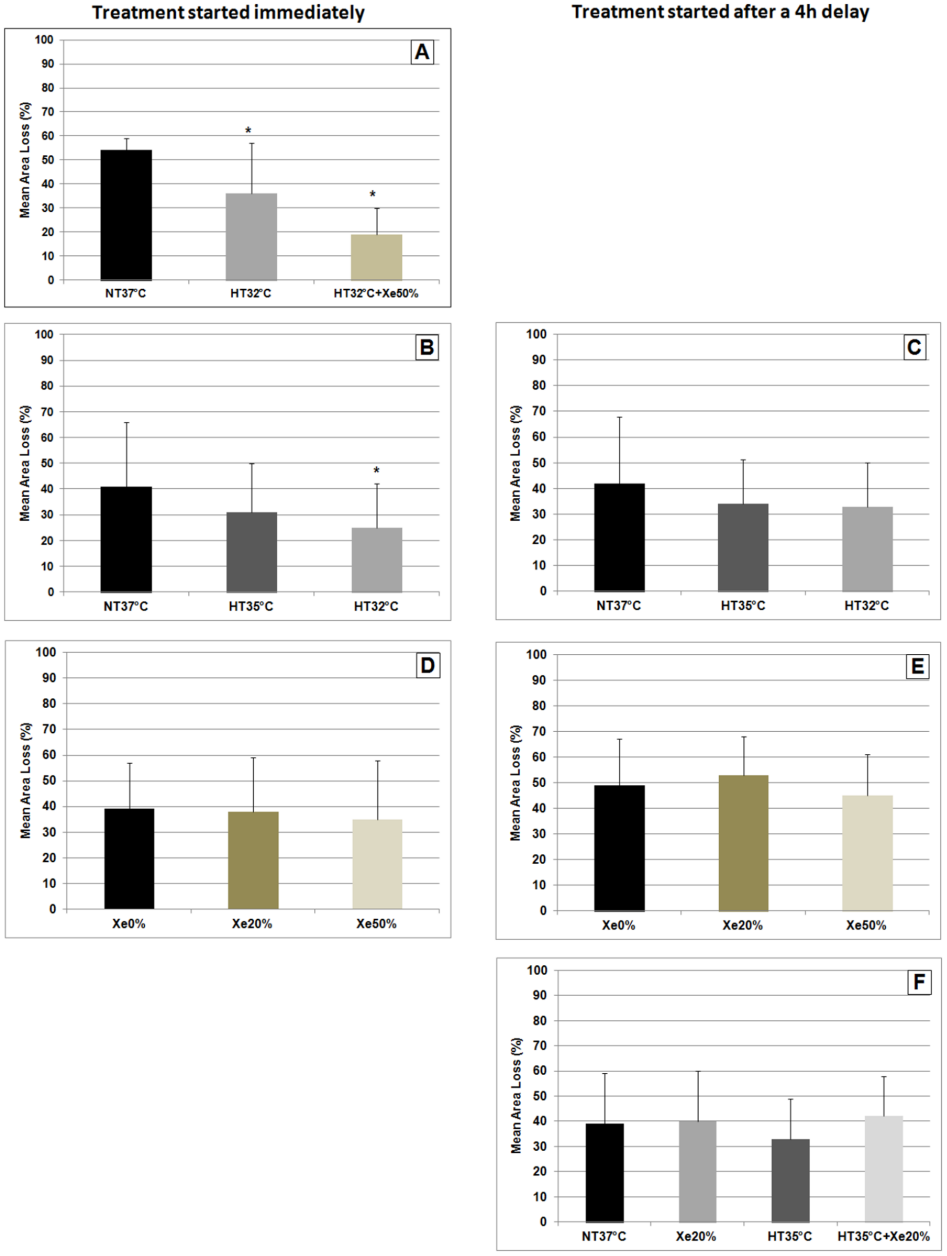
Shows the mean brain area loss (+SD) of each of the performed experiments, either being immediately after the hypoxic-ischemic insult or after a 4 h delay. (A) Showing significant neuroprotection of hypothermia (HT_32°C_) compared to normothermia (NT_37°C_) (p  =  0.02) and additional neuroprotection in the combination of 50% xenon with hypothermia (HT_32°C_+Xe_50%_) (p<0.001). (B+C) Shows a significant reduction of mean brain area loss in animals being immediately cooled to 32°C after hypoxia-ischemia, compared to animals being normothermic (p  =  0.04). Immediate hypothermia at 35°C (HT_35°C_) did not significantly reduce brain area loss (p  =  0.26). Starting the treatment with a 4 h delay showed no significant neuroprotection of both hypothermia temperatures compared to the normothermia group (p>0.05). (D+E) Shows that immediately after hypoxia ischemia neither 20% (Xe_20%_) nor 50% xenon (Xe_50%_) significantly reduce brain area loss compared to the group treated in air (Xe_0%_) (p>0.05). Starting the treatment with a 4 h delay did not change the results and showed a similar pattern (p>0.05). (F) Shows the results of the combined treatment started with a 4 h delay after hypoxia-ischemia. None of the treatment groups significantly reduced brain area loss compared to the NT_37°C_ group. The combination of HT_35°C_ and Xe_20%_started with a 4 h delay did not reduce brain area loss.
